# Assessment of the effect of potential antifibrotic compounds on total and αVβ6 integrin‐mediated TGF‐β activation

**DOI:** 10.1002/prp2.30

**Published:** 2014-06-09

**Authors:** Joanne Porte, Gisli Jenkins

**Affiliations:** ^1^ Division of Respiratory Medicine Nottingham University Hospitals Hucknall Road Nottingham NG5 1PB

**Keywords:** Idiopathic pulmonary fibrosis, integrin, transforming growth factor‐β

## Abstract

Transforming growth factor‐β (TGF‐β) plays an important role in the development of tissue fibrosis, and molecules inhibiting this pathway are attractive therapeutic targets for fibrotic diseases such as idiopathic pulmonary fibrosis (IPF). Activation of TGF‐β is the rate‐limiting step in TGF‐β bioavailability, and activation by the αVβ6 integrin is important in fibrosis of the lung, liver, and kidney. Activation of TGF‐β by αVβ6 requires direct cell–cell contact and measurable release of active TGF‐β in extracellular fluid compartments does not reflect tissue specific activation. The aim of this study was to determine the effect of antifibrotic compounds on both total, and specific αVβ6 integrin‐mediated TGF‐β activity. Using a transformed mink lung cell (TMLC) TGF‐β reporter, the effects of potential antifibrotic therapies including an activin‐like kinase (Alk5) inhibitor, Dexamethasone, Pirfenidone, *N*‐acetylcysteine (NAC), and BIBF1120 were assessed. Effects due to αVβ6 integrin‐mediated TGF‐β activity were measured using reporter cells cocultured with cells expressing αVβ6 integrins. These high‐throughput studies were validated using a phosphorylated Smad2 Enzyme‐Linked Immunosorbent Assay. Alk5 inhibitors are potent inhibitors of TGF‐β activity, whereas the novel antifibrotics, Pirfenidone, BIBF1120, and NAC are only moderate inhibitors, and Dexamethasone does not specifically affect TGF‐βactivity, but inhibits TGF‐β‐induced gene expression. None of the current small molecular inhibitors inhibit αVβ6‐mediated TGF‐β activity. These results demonstrate the potential of this high‐throughput assay of αVβ6‐specific TGF‐β activity and illustrate that currently available antifibrotics have limited effects on αVβ6 integrin‐mediated TGF‐β activity.

## Abstract 2

e00030

**Abbreviations**
Alk: activin‐like kinase
IPF: idiopathic pulmonary fibrosis
LAP: latency‐associated peptide
LTBPs: latent TGF‐β‐binding proteins
MEF: mouse embryonic fibroblast
NAC: *N*‐acetylcysteine
pSmad2: phosphorylated Smad2
TGF: transforming growth factor
FCS: foetal calf serum
TMLC: transformed mink lung cell
PAI‐1: plasminogen activator inhibitor‐1

## Introduction

Fibrotic diseases represent a significant, and escalating, burden globally, with fibrotic diseases of the lung, liver and kidney being responsible for 3.4% of the global mortality in 2010, having increased from 2.7% in 1990 (Lozano et al. 2012). Idiopathic pulmonary fibrosis (IPF) is an exemplar fibrotic disease. It is a chronic, progressive lung disease with a high mortality rate (Navaratnam et al. 2011), and at present, there is no effective therapy so patients progress to respiratory failure with a median survival of around 3 years (Gribbin et al. 2006). The incidence continues to rise with almost 5000 deaths per year in the United Kingdom. There is, therefore, an urgent unmet need for novel therapeutic interventions.

The cause of IPF remains unknown, but the current hypothesis for its pathogenesis proposes that repeated epithelial injury and a lack of adequate repair of the alveolar‐capillary basement membrane leads to infiltration of fibrogenic cells and subsequent excessive matrix production (Jenkins et al. 2012). Transforming growth factor‐β (TGF‐β) is a key cytokine that has been implicated in both epithelial repair and matrix deposition and there is considerable evidence that it plays a central role the pathogenesis of fibrotic diseases. Increased levels of TGF‐β have been found in fibroblastic foci of IPF patients (Broekelmann et al. 1991), overexpression of active TGF‐β induces fibrosis (Sime et al. 1997), whereas inhibition has prevented fibrosis (Wang et al. 1999) in animal models of disease. Despite the considerable evidence implicating TGF‐β in pulmonary fibrosis, no specific inhibitors of TGF‐β have emerged as therapies for IPF. TGF‐β1 is a member of the highly conserved TGF‐β superfamily, it is ubiquitously expressed and has pleiotropic effects on cells and organs throughout the body. Global inhibition of TGF‐β activity has potent proinflammatory effects, and TGF‐β inhibitors have been limited by their toxicity. It is likely that the pleiotropic effects of TGF‐β are mediated through tight spatial and temporal regulation of its activation.

TGF‐β1 is synthesized as a small latent complex in which the active TGF‐β is noncovalently associated with the latency‐associated peptide (LAP). This is then further associated with the latent TGF‐β‐binding proteins (LTBPs) to form the large latent complex, and through the LTBPs, is tethered to components of the extracellular matrix in its inactive form. Activation of TGF‐β requires dissociation from the LTBPs. There are different mechanisms by which activation can occur including physical effects such as extremes of heat or pH (Brown et al. 1990) and by proteolytic cleavage involving plasmin (Lyons et al. 1988), tryptase (Tatler et al. 2008), thrombin (Taipale et al. 1992), MMP's (Mu et al. 2002) or thrombospondin (Crawford et al. 1998). However, in vivo, a major mechanism of TGF‐β activation is via integrins (Munger et al. 1999).

The αVβ6 integrin is an epithelial cell‐restricted integrin that is expressed at low levels in normal lung but dramatically increased in fibrotic disease, and there is considerable evidence implicating this integrin in the pathogenesis of IPF (Munger et al. 1999; Horan et al. 2008; Puthawala et al. 2008; Xu et al. 2009), renal fibrosis (Ma et al. 2003; Hahm et al. 2007), and hepatic fibrosis (Wang et al. 2007; Popov et al. 2008; Sullivan et al. 2010). Therefore, inhibition of αVβ6 integrin‐mediated TGF‐β activation is an appealing strategy for the development of novel therapies in fibrotic disease, through targeting inhibition of TGF‐β activity to the site of disease thus avoiding significant “off‐target” effects.

This study used a high‐throughput reporter cell assay, and a phosphorylated Smad2 (pSmad2) Enzyme‐Linked Immunosorbent Assay (ELISA) to determine the total, and αvβ6 integrin‐specific, TGF‐β activity of four potential antifibrotic compounds, Pirfenidone, NAC, dexamethasone, and BIBF1120. Pirfenidone, NAC, and BIBF1120 were weak inhibitors of total TGF‐β activation, and dexamethasone had no intrinsic effect of TGF‐β activation but had moderate effects on TGF‐β signalling. These studies demonstrate an urgent need to develop novel anti‐αVβ6 integrin inhibitors.

## Materials and methods

### Cells and reagents

Mouse embryonic fibroblasts (MEFs) were previously stably transfected with the wild‐type β6 subunit (MEF‐β6) by this group (Xu et al. 2009). Cells were maintained in Dulbecco's Modified Eagle Medium (DMEM), 4 mmol/L l‐Glutamine, 10% foetal calf serum (FCS) containing 5 μg mL^−1^ Blastocidin (InVivoGen, San Diego, CA). The transformed mink lung reporter cells (TMLC) stably expressing firefly luciferase under the control of a TGF‐β‐sensitive portion of the plasminogen activator inhibitor‐1 (PAI‐1) promoter (Abe et al. 1994), were a gift from Dan Rifkin (New York University, New York), and were cultured in DMEM, 4 mmol/L l‐Glutamine, 10% FCS, and 250 μg mL^−1^ G‐418 sulphate (Sigma, Dorset, UK).

The antibodies used were mouse monoclonal anti αVβ6 ‐clone 10D5, (Millipore, Billerica, MA), F(ab')2 fragment of goat anti‐mouse IgG conjugated to R‐phycoerythrin (Life Technologies, Paisley, UK), and mouse monoclonal anti‐TGF‐β1, β2, β3 –clone 1D11 (R&D systems, Abingdon, UK).

The Alk5 inhibitor (SB525334A) was obtained from GSK (Stevenage, UK). Pirfenidone, NAC, and dexamethasone were purchased from Sigma (Dorset, UK) and BIBF1120 purchased from Selleckchem (Munich, Germany). TGF‐β1 was obtained from (R&D systems).

### Flow cytometry

Cells were harvested by trypsinization. Nonspecific interactions were blocked with goat serum for 20 min at 4°C. Cells were then washed in PBS containing Ca^2+^ and Mg^2+^ before incubating with the anti‐αVβ6 mouse monoclonal antibody, 10D5, at 10 μg mL^−1^ for 1 h at 4°C. After washing again, cells were incubated with goat anti‐mouse secondary antibody labelled with Phycoerythrin at a 1:200 dilution for 20 min at 4°C. Fluorescence was measured using a FACSCanto II flow cytometer (BD, Franklin Lakes, NJ), and analysed using FlowJo software (Treestar, OR).

### TMLC active TGF‐β assay

To measure the inhibition of αVβ6 mediated TGF‐β activity, a coculture of MEF cells stably transfected with the αVβ6 integrin was used. MEF‐β6 cells were seeded into 96‐well plates at a density of 2.5 × 10^5^ cells mL^−1^ in DMEM with 10% serum. The cells were incubated overnight to allow adherence. TMLC cells were harvested and resuspended at a concentration of 5 × 10^5^ cells mL^−1^ in DMEM + FCS, and any inhibitors or antibodies added to the correct concentration. Medium was removed from the MEF‐β6 cells and replaced with 100 μl of appropriate TMLC cell suspension. After 16 h incubation at 37°C, the cells were washed with PBS and then lysed with 50 μL of reporter lysis buffer (Promega, Hampshire, UK). The cell lysate was then transferred to a luminometry plate and 100 μL of luciferase assay reagent added (Promega). Luciferase activity was measured immediately using a FLUOstar omega multi‐mode microplate reader (BMG Labtech, Ortenburg, Germany).

To measure total active TGF‐β, TMLC cells were plated alone and serum starved overnight. A quantity of 2 ng mL^−1^ TGF‐β was added to each well with the appropriate inhibitor concentration and incubated for 16 h at 37°C. Harvest and measurement was identical to coculture experiments.

### Phosphorylated Smad2 ELISA

MEF‐β6 cells were plated in 10‐cm petri dishes and incubated at 37°C until 80% confluence. Inhibitors were added along with fresh medium, containing 10% FCS and 2 ng mL^−1^ TGF‐β. After 4 h of incubation at 37°C, nuclear protein was prepared using Nuclear Extract kit (Active Motif, La Hulpe, Belgium). Protein concentration was determined using BCA Protein Assay kit (Pierce, Rockland, IL). A quantity of 10 μg of nuclear protein was loaded into a Pathscan pSmad2 ELISA assay (Cell Signalling, Danvers, MA). Absorbance was read at 450 nm using a FLUOstar omega multi‐mode microplate reader (BMG Labtech).

### Statistical analysis

All values are expressed as mean ± SEM. The minimum number of replicates was three. Statistical analysis was performed using GraphPad Prism 6 software. Comparisons with control were performed using a one‐way analysis of variance (ANOVA) test. A *P *< 0.05 was considered significant.

## Results

### Currently available antifibrotic compounds are weak inhibitors of TGF‐β receptor signalling

**Figure 1:**
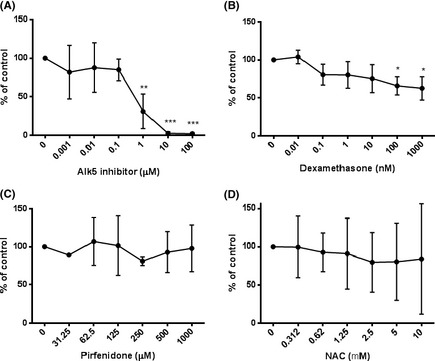
Various antifibrotic compounds were used to inhibit TGF‐β‐induced TMLC luciferase activity. SB525334 (A) lead to concentration‐dependent inhibition of TGF‐β‐induced TMLC reporter activation. Dexamethasone (B) lead to partial inhibition of the TGF‐β‐induced reporter activity in a concentration‐dependent manner. Pirfenidone (C) and NAC (D) had no effect. All experiments were performed in triplicate and repeated three times. Data presented are the mean of three independent experiments and expressed as a percentage of untreated controls. Data expressed as mean ± standard error. *P < 0.05, **P < 0.01, ***P < 0.001.

**Figure 2:**
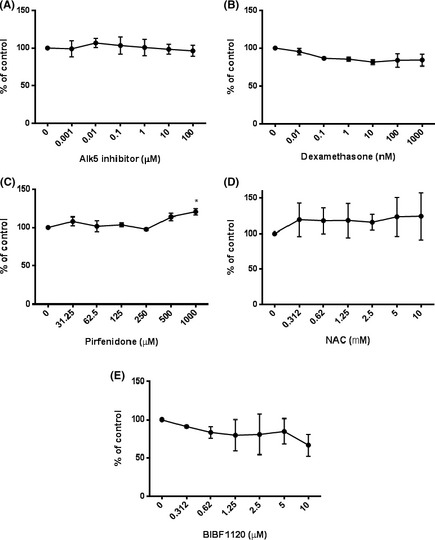
Antifibrotic compounds were not toxic to TMLC reporter cells. SB525334 (A) Dexamethasone (B) Pirfenidone (C), and NAC (D) had no effect on levels of cell death of TMLC reporter cells. (E) BIBF1120 did increase cell death in a concentration‐dependent manner. All experiments were performed in triplicate and repeated three times. Data presented are the mean of 3 independent experiments and expressed as a percentage of untreated controls. Data expressed as mean ± standard error.

To determine the effect of potential antifibrotic compounds on TGF‐β activity and signalling TMLCs were used. TMLCs were stimulated with exogenous TGF‐β in the presence of a small molecular Alk5 inhibitor (SB525334A), Dexamethasone, Pirfenidone, BIBF1120, and NAC. SB525334A inhibited TGF‐β activity with an IC_50_ of ~0.5 μmol/L (Fig. 1A). Dexamethasone was a weak inhibitor of TGF‐β activity with maximal inhibition of TGF‐β induced reporter activity of between 20% and 50%. Dexamethasone inhibited TGF‐β induced reporter activity in the nanomolar range (Fig. 1B), Pirfenidone and NAC had no effect on TGF‐β reporter activity (Fig. 1C and D). None of the concentrations used were toxic to TMLC cells (Fig. 2A–D). BIBF1120 did appear to inhibit TGF‐β‐induced reporter cell activity but this was due to toxic effects on the reporter cells (Fig. 2E).

### Total cellular TGF‐β activation can be discriminated from αVβ6 integrin‐specific activation using an αVβ6 expressing and TMLC reporter coculture assay

**Figure 3:**
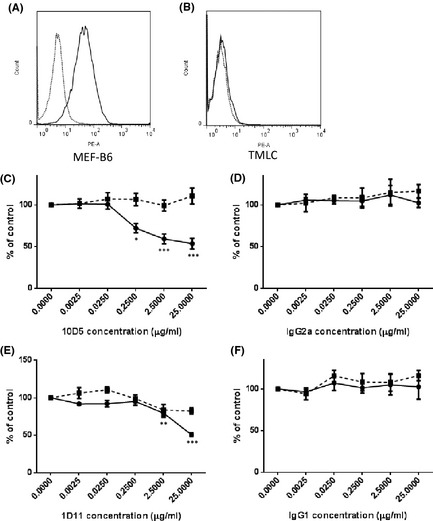
Cell surface αVβ6 integrin is expressed at high levels on MEF cells stably transfected with the pWZL‐ β6 plasmid (A), but not TMLC reporter cells (B). Solid line = 10 mg mL^−1^ 10D5 antibody. Dotted line = secondary antibody only. The integrin αVβ6‐neutralizing antibody 10D5 lead to a concentration‐dependent reduction in luciferase activity in coculture, without effecting levels of luciferase expression from TMLC reporter cells alone (C). An IgG2a isotype control antibody had no effect in either system (D). The pan‐isoform TGF‐β neutralizing antibody 1D11 lead to partial inhibition of luciferase activity from both coculture and TMLC reporter cells (E). IgG1 Isotype control antibody had no effect on either system (F). Solid line = coculture. Dotted line = TMLC cells only. All experiments were performed in triplicate and repeated three times. Data presented are the mean of three independent experiments and expressed as a percentage of untreated controls. Data expressed as mean ± standard error. *P < 0.05, **P < 0.01, ***P < 0.001.

**Figure 4:**
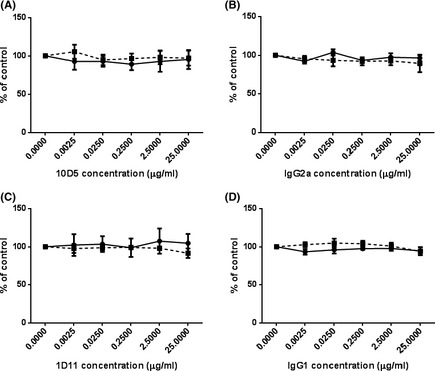
Neutralizing antibodies were not toxic to TMLC reporter cells. 10D5 (A) IgG2a control (B) 1D11 (C) IgG1 (D) had no effect on levels of cell death of TMLC reporter cells. Solid line = coculture. Dotted line = TMLC cells only. All experiments were performed in triplicate and repeated three times. Data presented are the mean of three independent experiments and expressed as a percentage of untreated controls. Data expressed as mean ± standard error.

To determine whether antifibrotic compounds were able to inhibit total cellular TGF‐β or αVβ6 integrin‐specific TGF‐β activation, a coculture assay using an αvβ6 integrin‐expressing cell line and the TMLC reporter cell line were used. High levels of the αVβ6 integrin were confirmed on the experimental cells and the absence of this integrin was confirmed on TMLCs by flow cytometry (Fig. 3A and B). To confirm the coculture assay could selectively distinguish between αVβ6 mediated TGF‐β and total cellular TGF‐β activation, the αVβ6 specific blocking antibody 10D5 and the pan‐TGF‐β blocking antibody 1D11 were used. 10D5 lead to concentration‐dependent inhibition of the coculture with an IC_50_ of 0.25 μg mL^−1^, without effecting TMLC reporter cells at any concentration tested (Fig. 3C) . Similarly, an IgG2a control had no effect on either the coculture or the reporter cells alone (Fig. 3D). Surprisingly, 1D11 had no effect on either the coculture or the reporter cells at concentrations below 2.5 μg mL^−1^ (Fig. 3E), but at a concentration of 25 μg mL^−1^ a 50% inhibition was observed in the coculture. The IgG1 control had no effect on either the coculture or the reporter cells (Fig. 3F). None of the antibodies used had toxic effects on either the reporter cells or the coculture (Fig. 4).

### Currently available antifibrotic compounds are poor inhibitors of αVβ6‐mediated TGF‐β activation

**Figure 5:**
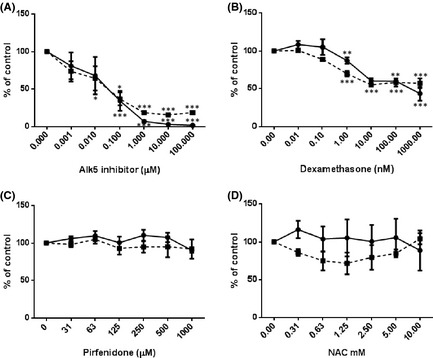
αVβ6‐dependent TGF‐β activity was determined by coculturing MEF‐β6 cells with TMLC reporter cells in the presence of inhibitors, effects on total cellular TGF‐β activity were determined by culturing TMLC reporter cells in the presence of inhibitors alone. SB525334 lead to a concentration‐dependent decrease in both total cellular, and αVβ6 integrin‐dependent, luciferase activity (A). Dexamethasone had a similar but less potent effect on both total, and αVβ6 integrin‐dependent luciferase activity (B). Pirfenidone had no effect on total cellular TGF‐β activity and no effect on αVβ6 integrin‐dependent luciferase activity (C). NAC at low concentrations had marginal effects on total cellular TGF‐β, which decreased at higher concentrations and no effect on αVβ6 integrin‐mediated TGF‐β (D). Solid line = coculture. Dotted line = TMLC cells only. All experiments were performed in triplicate and repeated three times. Data presented are the mean of three independent experiments and expressed as a percentage of untreated controls. Data expressed as mean ± standard error. *P < 0.05, **P < 0.01, ***P < 0.001.

Having confirmed that the coculture assay could distinguish between total and αVβ6 integrin‐specific TGF‐β activity, the potential antifibrotic compounds SB525334A, dexamethasone, Pirfenidone, and NAC were assessed. SB525334 and Dexamethasone inhibited both reporter cells and coculture in a concentration‐dependent manner with an IC_50_ of 5 nmol/L and 1 nmol/L respectively (Fig. 5A and B) suggesting inhibition of total cellular, rather than αvβ6‐specific TGF‐β activity. Pirfenidone and NAC had no effect on αVβ6‐dependent or total cellular TGF‐β activation. (Fig. 5C and D).

### Currently available antifibrotic compounds are moderate inhibitors of Smad2 phosphorylation

**Figure 6:**
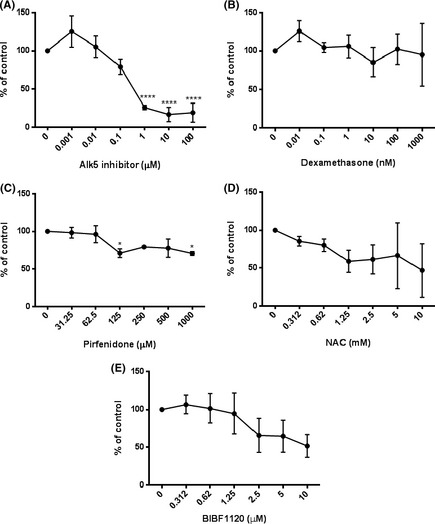
To confirm the effects on reporter cells MEF‐β6 cells were cultured in the presence of inhibitor for 4 h and pSmad2 was measured by ELISA. SB525334 lead to a concentration‐dependent inhibition of pSmad2 (A), Dexamethasone had no effect on pSmad2 (B). Pirfenidone lead to moderate concentration‐dependent inhibition of pSmad2 (C). NAC had variable and nonsignificant inhibition of pSmad2 (D). BIBF1120 lead to concentration‐dependent inhibition of pSmad2 at high concentration (E). All experiments were performed in triplicate and repeated three times. Data presented are the mean of three independent experiments and expressed as a percentage of untreated controls. Data expressed as mean ± standard error. *P < 0.05, **P < 0.01, ***P < 0.001.

The most proximal step following TGF‐β receptor ligation is phosphorylation of the receptor Smad2. To determine the effect of the potential antifibrotic compounds on TGF‐β activation and receptor inhibition an ELISA measuring levels of pSmad2 was used. SB525334A inhibited pSmad2 in a concentration‐dependent manner with an IC_50_ of 100 nM (Fig. 6A) similar to previous data using the coculture assay (Figs. 1A and 5A). In contrast, dexamethasone had no effect on pSmad2 levels (Fig. 6B), demonstrating that all effects on reporter cell assays (Fig. 1B and 5B) were due to direct effects on the PAI1 promotor, not through inhibition of TGF‐β activation or receptor ligation. Pirfenidone, BIBF1120, and NAC had moderate but variable effects on pSmad2 levels (Fig. 6C and D) supporting observations in coculture assays.

## Discussion

TGF‐β activation and signalling pathways are central to the pathogenesis of fibrosis. However, the determination of biologically relevant TGF‐β in experimental systems is challenging due to the tight spacial and temporal regulation of TGF‐β activation in tissues. Activation of TGF‐β by the αVβ6 integrin is likely to be a central driver of lung fibrosis, but measurement of αVβ6 integrin‐mediated TGF‐β activation is especially challenging because it does not lead to free release of measurable amounts of active TGF‐β. Assays that rely entirely on measuring TGFβ in the supernatants of biological fluids will give unreliable assessments of TGFβ activity because they do not take into account cell‐associated activation of TGFβ. Furthermore, because total TGFβ is synthesized in considerable excess of biologically active TGFβ (Xu et al. 2009), and activation of TGFβ is the rate‐limiting step in TGFβ bioavailability (Annes et al. 2003) assays that measure synthesis of TGFβ at the message level will give little indication of its bioavailability. Therefore, we sought to systematically assess the ability of currently clinically available antifibrotic compounds to inhibit cell‐free, total cellular, and αvβ6 integrin‐mediated TGFβ activity in cell‐based assays.

We use an in vitro model utilizing MEFs stably transfected with wild‐type β6 integrins. Although the αvβ6 integrin is restricted to epithelial cells in vivo, this system has a number of advantages for measuring αvβ6‐mediated TGFβ activation in vitro. Although primary epithelial cells express high levels of αvβ6 integrins, expression is lost in epithelial cell lines, therefore, MEF‐β6 cells offer a high level of uniform αvβ6 expression in rapidly proliferating cells. Furthermore, αvβ6 integrin‐mediated TGFβ activation relies on cellular contraction (Shi et al. 2011), and fibroblasts are more contractile than epithelial cells, thus the measurable signal is amplified. Our data demonstrate a rational, and practical, strategy to determine the relative contribution of total and αvβ6 integrin‐specific TGF‐β activation using in vitro assays that can be used to assess the preclinical potential of antifibrotic molecules.

SB525334A is an Alk5/type 1 TGF‐β receptor kinase inhibitor that inhibits Alk5 phosphorylation of Smad3 with an IC_50_ of 14 nmol/L in kinase assays (Grygielko et al. 2005). Furthermore, it inhibits TGF‐β induced PAI1 mRNA and procollagen gene synthesis with an IC_50_ in the nanomolar range (Grygielko et al. 2005). It is reassuring, if unsurprising, that inhibition of physiological concentrations of cell‐associated TGF‐β occurred with SB525334A in the nanomolar range, consistent with these previous observations. Although SB525334A inhibits experimental models of pulmonary artery hypertension (Thomas et al. 2009), renal fibrosis (Grygielko et al. 2005), and mesenchymal tumours, it has antiapoptotic and mitogenic effects in epithelial cells raising concerns regarding potential toxicity that may limit its therapeutic utility (Laping et al. 2007).

Somewhat surprisingly, the pan‐TGF‐β‐isoform neutralizing antibody 1D11 did not completely inhibit total cellular, or αVβ6 integrin‐mediated TGF‐β activity at concentrations below 2.5 μg mL^−1^. Original descriptions of 1D11 demonstrate that it is able to inhibit the growth inhibitory activity of both TGF‐β1 and TGF‐β2 at concentrations of 10 μg mL^−1^ (Dasch et al. 1989). Although our data would confirm that this concentration of 1D11 is sufficient to inhibit TGF‐β activity, a comparison of its TGF‐β inhibitory effect in relation to other inhibitors has not previously been performed, and it is apparent that it has fairly low‐level TGF‐β inhibitory activity. This is probably due to the mechanism of action of TGF‐β activity in vitro and in vivo, which in many circumstances, including activation by αVβ6 integrins, requires cytoplasmic forces to alter the structure of the latent TGF‐β complex that is tethered to the extracellular matrix (Fontana et al. 2005; Wipff et al. 2007). The active TGF‐β is not released into the extracellular fluid in large quantities because cells have to be in direct cell–cell contact to respond to the TGF‐β (Munger et al. 1999). Thus, it is likely that the steric hindrance provided by the matrix‐latent TGF‐β complex is sufficient to inhibit the effect of 1D11 in physiological conditions, and this may explain the disappointing effects of humanized TGF‐β‐neutralizing antibodies in clinical trials of fibrotic disease (Denton et al. 2007).

The αVβ6‐neutralizing antibody, 10D5, did not completely inhibit the reporter system. However, it is able to inhibit TGF‐β activity in the coculture at concentrations as low as 0.25 μg mL^−1^ consistent with previous reports (Weinreb et al. 2004), and has no effect on reporter cells that don't express αVβ6 integrins demonstrating its specificity for αVβ6 mediated pathways. The lack of complete inhibition may reflect steric hindrance, alternative pathways of TGF‐β activation, and possibly the presence of active TGF‐β within the cell culture medium. We have observed similar effects in serum‐free conditions, and although other αVβ6‐neutralizing antibodies are considerably more potent in the same system (Weinreb et al. 2004), they were also unable to completely neutralize TGF‐β activity. This suggests that alternative TGF‐β activation pathways, possibly via TGF‐β2 (Neurohr et al. 2006), are playing a role in homeostatic TGF‐β activation.

**Figure 7:**
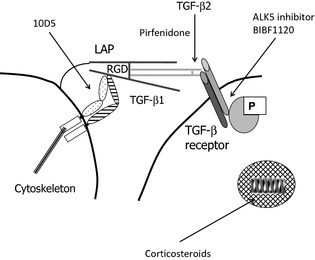
αVβ6 integrins constitutively bind the TGF‐β1‐latency‐associated peptide (LAP) to homeostatically activate epithelial TGF‐β, which then acts on neighbouring cells that are in direct cell–cell contact. Schematic identifying potential sites of action for potential novel antifibrotic therapies.

Pirfenidone is a promising antifibrotic molecule licensed for the treatment of IPF (Jenkins 2013). The precise mechanism of Pirfenidone's action remains unknown but it has been described as an inhibitor of TGFβ signalling and activity (Cho and Kopp 2010) and is thought to exert its clinical effects through inhibition of TGFβ production (Azuma 2012). It can inhibit TGF‐β gene expression in the lungs of bleomycin‐treated hamsters (Iyer et al. 1999), and kidneys of cyclosporine‐treated rats (Shihab et al. 2002), furthermore, it can inhibit TGF‐β2 protein and mRNA expression in malignant human cells (Burghardt et al. 2007). However, no studies have systematically assessed the effect of Pirfenidone on TGFβ activation. Our studies have used concentrations of Pirfenidone that are biologically relevant, similar to levels observed in plasma from patients (Shi et al. 2007; Rubino et al. 2009) and would suggest that Pirfenidone has no TGF‐β1,3 inhibitory activity in the range of 30–1000 μmol/L (equivalent to 5.8–185 μg mL^−1^). Pirfenidone has been previously shown to inhibit TGF‐β1‐induced responses in human lung cells at between 100 and 500 μg mL^−1^ (Nakayama et al. 2008; Hisatomi et al. 2012) and it has been shown to specifically inhibit TGF‐β2‐induced Smad3 nuclear translocation in lens epithelial cells at 500 μg mL^−1^ (Yang et al. 2013). Our studies would suggest that physiological concentrations of Pirfenidone have no effect on αVβ6 integrin‐mediated TGF‐β activation, which is the predominant pathway for TGF‐β1 activation in the alveoli. We cannot exclude the possibility that Pirfenidone preferentially inhibits TGF‐β2 activation pathways that are independent of the αvβ6 integrin (see Fig. 7). Therefore, Pirfenidone in combination with αvβ6‐neutralizing strategies may be beneficial as combination therapies in fibrotic disease targeting different aspects of TGFβ signalling pathways.

Reactive oxygen species (ROS) are thought to play a significant role in fibrogenesis (Liu and Gaston Pravia 2010) and NAC is used for its antioxidant properties as a therapy in IPF (Demedts et al. 2005). ROS is thought to specifically activate latent TGF‐β1 (Jobling et al. 2006), and NAC has been shown to inhibit Smad3 phosphorylation in lung epithelial cells (Felton et al. 2009). Although there are no data to suggest oxidant‐mediated injury plays a role in αvβ6 integrin‐mediated TGFβ activation, the role of NAC in αVβ6 integrin‐mediated TGF‐β activation has not been studied. The concentrations of NAC used in this study range from the physiological, 0.3 mmol/L, (Borgstrom et al. 1986) to previously used in vitro concentrations, 5 mmol/L (Felton et al. 2009) and 10 mmol/L (Lavrentiadou et al. 2001). In contrast with the studies by Felton et al. (2009), our data do not suggest that NAC has consistent TGF‐β inhibitory activity. This may be because the previous studies used a prolonged culture protocol to assess effects of NAC on EMT. Our data do suggest a trend towards inhibition of pSmad2 at high concentrations of NAC, but these concentrations are of uncertain biological relevance and do not support a role for NAC in αvβ6 integrin‐mediated TGFβ activation.

Dexamethasone inhibited TGF‐β‐stimulated, cell‐associated, and αvβ6 integrin‐mediated reporter activities; however, it had no effect on Smad2/3 phosphorylation. These data confirm the known effect of ligated glucocorticoid receptor, namely that it inhibits transcriptional activation of the PAI‐1 promoter by Smad3 (Song et al. 1999), but demonstrate that dexamethasone has no effect on TGF‐β activation pathways (see Fig. 7).

BIBF 1120 is highly selective for angiogenic tyrosine kinase receptors with an IC_50_ in the nanomolar range for vascular endothelial growth factor, platelet derived growth factor, and fibroblast growth factor in kinase assays and can inhibit ligand induced mitogen‐activated protein kinase and Akt phosphorylation at 0.3 μm L^−1^ (Hilberg et al. 2008). Alk5 is a serine/threonine kinase receptor and thus it was not anticipated that BIBF1120 would have any effect on TGFβ signalling. However, at high concentrations BIBF1120 did show a trend towards inhibiting TGFβ activity by coculture and pSmad2 assays. Although BIBF 1120 had toxic effects on TMLC reporter cells at moderate concentrations, it was tolerated by experimental cells. Furthermore, the pSmad2 ELISA is adjusted for protein content, limiting the confounding effects of cellular toxicity, suggesting that the effects of BIBF 1120 on pSmad2 are not due to generalized cytopathic effects. However, the concentrations required to effect TGFβ pathways are much higher than therapeutic concentrations (Mross et al. 2010), suggesting that BIBF1120 is unlikely to have any clinical effect via TGF‐β signalling.

These data demonstrate that current antifibrotic therapies have, at best, marginal effects on TGF‐β activation at therapeutic concentrations. Although these data are not entirely unexpected, they are important because TGFβ activation and signalling pathways are central in the pathogenesis of IPF. Furthermore, these data highlight practical, and robust preclinical assays for determining total and αVβ6 integrin‐mediated TGF‐β activation. Given the likely importance of αvβ6 integrin‐mediated TGF‐β activation in the development of fibrogenesis there is a clear unmet need for further development of molecules that target this pathway. These assays will be useful preclinical assessment tools in the development of such compounds.

## Disclosure

G. Jenkins has received consultancy income from GlaxoSmithKline, Intermune, and Boehringer Ingelheim. He has received lecture fees from Intermune, MedImmune, and Boehringer Ingelheim. He has sponsored research agreements with GlaxoSmithKline, Novartis, and Biogen Idec.
